# Analyzing differences between restricted mean survival time curves using pseudo-values

**DOI:** 10.1186/s12874-022-01559-z

**Published:** 2022-03-18

**Authors:** Federico Ambrogi, Simona Iacobelli, Per Kragh Andersen

**Affiliations:** 1 Department of Clinical Sciences and Community Health, University of Milan, Milan, Italy; 2 Scientific Directorate, IRCCS Policlinico San Donato, Milan, Italy; 3 Department of Biology, University of Rome Tor Vergata, Rome, Italy; 4 Department of Biostatistics, University of Copenhagen, Copenhagen, Denmark

**Keywords:** RMST curve difference, Pseudo-values, Crossing survival curves

## Abstract

Hazard ratios are ubiquitously used in time to event analysis to quantify treatment effects. Although hazard ratios are invaluable for hypothesis testing, other measures of association, both relative and absolute, may be used to fully elucidate study results. Restricted mean survival time (RMST) differences between groups have been advocated as useful measures of association. Recent work focused on model-free estimates of the difference in restricted mean survival through follow-up times, instead of focusing on a single time horizon. The resulting curve can be used to quantify the association in time units with a simultaneous confidence band. In this work a model-based estimate of the curve is proposed using pseudo-values allowing for possible covariate adjustment. The method is easily implementable with available software and makes possible to compute a simultaneous confidence region for the curve. The pseudo-values regression using multiple restriction times is in good agreement with the estimates obtained by standard direct regression models fixing a single restriction time. Moreover, the proposed method is flexible enough to reproduce the results of the non-parametric approach when no covariates are considered. Examples where it is important to adjust for baseline covariates will be used to illustrate the different methods together with some simulations.

**Supplementary Information:**

The online version contains supplementary material available at (10.1186/s12874-022-01559-z).

## Introduction

In most clinical trials and observational studies dealing with time-to-event as the main outcome, the measure of association used is the hazard ratio (HR), a quantity which is typically estimated using Cox regression [1]. When the proportional hazards assumption holds, Cox regression is, in fact, the preferred method of estimation due to its efficiency. The use of hazard ratios is well established and accepted in biomedical literature, sometimes acritically. In fact, many authors warned against its limitations. First of all, its interpretation may not always be as straightforward as could be a time based measure [2]. This is in part due to the relative nature of the hazard ratio, which means that the time gained by treated/exposed versus non-treated/non-exposed patients is not easily evaluated as it depends also on the baseline risk. Second, if the PH assumption is not true, reporting a single HR estimate is obviously misleading while the reporting of an HR varying through time does not have a simple interpretation due to selection of patients during follow-up [3, 4]. In some situations, the proportional hazards assumption is tenable just because of the fact that the follow-up length is too short to show non proportionality [5]. The need for expressing study results in a way that people can easily understand [6] is another of the motivations that keep the debate on the hazard ratio active.

Existing proposed alternatives include the ratio between median survival times [2], the difference of survival probabilities at a specific time point, and the difference of the expected survival times [7, 8]. The latter measure of association is in general referred to a fixed time interval [0,*τ*], i.e. the question is if there is a difference in the restricted (at *τ* years) mean survival time (RMST) [9]. In the framework of clinical trial planning, the comparison of RMST has interesting advantages [10].

An extreme form of non PH is when the survival curves cross and in such a situation a single measure of association, such as the hazard ratio, is too simple to summarise the relationship between the exposure or the treatment and the outcome. The restricted mean survival time (RMST) has been advocated as a possible alternative outcome measure for such cases [8, 10].

A possibility, introduced first by Royston and Parmar (2011) [8], is to estimate the treatment difference in RMST through the follow-up time, to show how the treatment comparison varies in time. This approach was developed non-parametrically introducing the use of simultaneous confidence bands to make inference at all time points [11]. This approach could be especially useful for investigating equivalence or non-inferiority questions.

In this work, we propose a simple, model-based, method to estimate the difference in RMST curves using pseudo-values. Such a methodology enables an easy calculation of the confidence bands for the curve and also the possibility of adjusting for covariates.

The use of pseudo-values is not the only possibility. The flexible parametric survival models [8] or the direct regression method [12], based on weighted estimating equations, are valid alternatives. Although software is readily available for all the cited approaches [8, 13, 14], the approach developed here, based on pseudo-values, allows an easy estimation of simultaneous confidence bands by means of available standard software.

Three applications are presented where adjustment by covariates is needed, together with a simulation study taking into account different scenarios.

## Methods

In this section we will present: 1) the definition of the RMST; 2) the general regression model on pseudo-values; 3) the modelling of the RMST with right-censored failure time data with covariates using pseudo-values through a linear model; 4) the extension to smoothing functions and time-varying effects; 5) the model-based comparison of RMST curves.

### Restricted mean survival time

In survival analysis the time *T* elapsed from an initial event to the possible occurrence of a terminating event is analysed. Usually, only a right-censored version of the random variable *T* is observed and, therefore, the mean value of *T* is not easy to estimate non-parametrically, [15]. As a replacement, the *τ*-restricted mean survival time (RMST) is defined as:

1 $$ \text{RMST}(\tau) = \int_{0}^{\tau} \mathrm{S}(t) dt   $$

where $\mathrm {S}(t) = \mathrm {P}(T>t) = \exp (- \int _{0}^{t} \lambda (u) du)$ is the survival function and *λ*(*t*) is the hazard function. The RMST(*τ*) represents the expected lifetime, *E*(*T*∧*τ*) over a time horizon equal to *τ*. The difference between RMST(*τ*) for different treatments has been advocated as a useful summary measure in clinical applications [7, 8, 10].

### General regression model on pseudo-values

The idea of using pseudo-values for censored data analysis was introduced by Andersen et al. (2003) [16]. Let *X*_*i*_,*i*=1,…,*n*, be independent and identically distributed random variables and *θ* be a parameter of the form

2 $$ \theta = E(f(X_{i}))  $$

and assume that we have an (at least approximately) unbiased estimator, $\hat {\theta }$, for this parameter. Let ***Z***_*i*_,*i*=1,…,*n* be independent and identically distributed covariates and define the conditional expectation

3 $$ \theta_{i} = E(f(X_{i}) | \boldsymbol{Z}_{i})  $$

The *i*^*th*^ pseudo-observation is defined as

4 $$ \hat{\theta}_{i} = n \hat{\theta} - (n-1) \widehat{\theta^{-i}}  $$

where $\widehat {\theta ^{-i}}$ is “the leave-one-out”estimator for *θ* based on *X*_*j*_,*j*≠*i*. If all *X*_*i*_ are observed then *θ* may be estimated by the average of the *f*(*X*_*i*_) in which case $\hat {\theta }_{i}$ is simply *f*(*X*_*i*_). This approach will be used here with a censored sample of the *X*_*i*_. A regression model for the parameter *θ* corresponds to a specification of how *θ*_*i*_ depends on *Z*_*i*_ and this may done via a generalized linear model

5 $$ g({\theta}_{i}) = \boldsymbol{\beta}^{T} \boldsymbol{Z}_{i}  $$

where the matrix ***Z*** contains a column of 1, corresponding to the intercept. The regression coefficients ***β*** can be estimated using generalized estimating equations [17]

6 $$ \begin{aligned} U(\boldsymbol{\beta}) = \sum_{i=1}^{n} U_{i}(\boldsymbol{\beta}) = \sum_{i=1}^{n} \left(\frac{\partial}{\partial \boldsymbol{\beta}} g^{-1} (\boldsymbol{\beta}^{T} \boldsymbol{Z}_{i}) \boldsymbol{R}_{i}^{-1} (\hat{\theta}_{i} - g^{-1} (\boldsymbol{\beta}^{T} \boldsymbol{Z}_{i})) \right)  \end{aligned}  $$

In the general situation *θ* may be multivariate and ***R***_*i*_ is a working covariance matrix [18]. Andersen et al. (2003) [16] argued that the variances of ***β*** can be obtained by the standard sandwich estimator

7 $$ \hat{\boldsymbol{V}} = I(\hat{\boldsymbol{ \beta}})^{-1} \hat{var} \{U(\boldsymbol{\beta}) \} I(\hat{\boldsymbol{ \beta}})^{-1}  $$

where

8 $$ I(\boldsymbol{ \beta}) = \sum_{i=1}^{n} \left(\frac{\partial g^{-1}(\boldsymbol{\beta}^{T} \boldsymbol{Z}_{i})}{\partial \boldsymbol{\beta}} \right)^{T} \boldsymbol{R}_{i}^{-1} \left(\frac{\partial g^{-1} (\boldsymbol{\beta}^{T} \boldsymbol{Z}_{i})}{\partial \boldsymbol{\beta}} \right)  $$

9 $$ \hat{var} \{U(\boldsymbol{\beta}) \} = \sum_{i=1}^{n} U_{i}(\boldsymbol{\beta}) U_{i}(\boldsymbol{\beta})^{T}  $$

After the computation of pseudo-values, parameter estimates and their standard errors can be obtained using standard statistical software for generalized estimating equations, though the standard errors may be slightly conservative. In fact, a general asymptotic theory of estimates from estimating functions based on pseudo-values demonstrated, under some regularity conditions, consistency and asymptotic normality of the estimates [19]. The ordinary sandwich estimator is however not consistent, leading to an overestimate of the standard errors. The demonstration is derived for real-valued pseudo-values, but, as stated in [19], it can be generalized to handle vector-valued pseudo-values.

Given the asymptotic normality of the model estimates, it is possible to adopt the approach based on simultaneous inference in general parametric models to obtain simultaneous test procedures and confidence intervals [20].

### Modelling of the RMST with right-censored failure time data with covariates using pseudo-values through a linear model

The RMST(*τ*) can be estimated non-parametrically based on the Kaplan-Meier estimator [1] or model-based, possibly resorting to flexible regression.

A model estimate for the RMST can be obtained, through transformation, adopting a model for the hazard function. The piecewise-exponential model assumes proportionality for the covariate effects while separate, piecewise constant, baseline hazards, are used for the different treatments to estimate RMST(*τ*) [21]. The model was further developed using Cox regression with stratification [22]. The method is implemented in the function restricted.residual.mean in the package timereg [23], in the free R software, [24]. The function can use the Cox regression model or the Aalen regression model to perform the calculations.

A number of different alternatives are available for model based estimates of RMST(*τ*), for example one convenient possibility is the use of flexible parametric survival models [8]. In general, the standard errors of the RMST (or of the difference of RMST between treatments) are obtained using the delta method, or using the bootstrap or other resampling/simulation techniques.

An alternative estimation method is to directly model RMST as a function of covariate values. This can be achieved using inverse probability of censoring weighting [12], or with pseudo-values [25]. We will focus on estimation based on pseudo-values as it allows to use standard software for generalized linear models and for simultaneous inference in general parametric models.

For the restricted mean we have $\theta = E(X \wedge \tau) = \int _{0}^{\tau } S(t) dt$, and we use the estimator obtained by plugging in the Kaplan-Meier estimator [25]. For this estimator, results stated in [19] are valid under the assumption of censoring independent of event times and covariates. The *i*^*th*^ pseudo-value at time *τ* is therefore defined as:

10 $$ \widehat{\theta}_{\tau \, i} = n \int_{0}^{\tau} \widehat{\mathrm{S}}(t) dt - (n-1) \int_{0}^{\tau} \widehat{\mathrm{S}}^{-i}(t) dt  $$

where $\widehat {\mathrm {S}}^{-i}(t)$ is the Kaplan-Meier estimator excluding subject *i*.

Instead of considering a single *τ*, as in [25], we consider a finite grid of *M* time points *τ*_1_,…,*τ*_*j*_,…,*τ*_*M*_ and we compute the pseudo-values for the *i*^*th*^ subject at each *τ*_*j*_. Time points can be selected as quantiles of the event time distribution, e.g. *M* could be chosen to have approximately 10 events for each pseudo-value while, in general, it is not useful to have more than 15-20 time points. In fact according to [13, 26] a number of points between 5 and 10 is sufficient to provide good estimates of the regression parameter. In [16], when studying the application to multi state models, model results using 5, 10 or 20 time points produced similar results. The problem with using a large number of time points is the computational burden as the dataset is being expanded with as many subjects’ replications as the number of time points considered.

A regression model for a vector valued $\hat {\boldsymbol {\theta }}_{i}$, with components calculated at several *τ*-values, must include terms for time and possibly interaction terms between covariates and time to account for time-varying covariate effects. This was already done for model based on pseudo-values with applications to competing risks [26, 27]. A generalized linear model can be assumed as:

11 $$ \mathrm{g}(\theta_{\tau_{j} \, i}) = \alpha_{j} + \mathbf{\gamma}^{T} \mathbf{C}_{i}   $$

where *α*_*j*_ determines the baseline (transformed) restricted mean time at *τ*_*j*_ and is explicitly estimated and ***C***_*i*_ are time-independent covariates.

A presentation of the different methods available for estimating RMST, applied to the classical Freireich data [28], can be found in the supplementary material [see Additional file 1].

### Extension to smoothing functions and time-varying effects

The baseline estimate for the grid points can be modelled by including *M*−1 dummy variables. It is possible to include time-varying effects through interaction terms between time and covariates. To include a reasonable number of regressors into the model, we propose to use a smoothing function (e.g. a cubic spline) to model the restricted mean time of the reference category. In such a way, model (11) can be rewritten as:

12 $$ g[\theta_{\tau_{j} \, i}]=\mathbf{\delta }^{T} \mathbf{B}_{j}+\mathbf{\gamma}^{T} \mathbf{C}_{i} +\mathbf{\zeta}^{T} \mathbf{b}_{B_{j} C_{i}} = \mathbf{\beta}^{T}\mathbf{Z}   $$

where **B**_*j*_ is the vector of the spline basis functions and **δ**^*T*^ the corresponding vector of the regression coefficients and $\mathbf {b}_{B_{j} C_{i}}$ represents the interaction terms among covariates and the spline bases to account for time-varying effects.

When considering the identity link, as covariate effects estimate differences in RMST, a constant difference through time is not plausible. Covariates should then be included together with their time-dependent effects, i.e. interactions with time.

### Model-based comparison of RMST curves

In the following the identity link will be used and, for ease of notation, only a binary covariate, *A*, will be considered. The regression model will therefore be specified as:

13 $$ \text{RMST}(\tau_{j}\mid A_{i}) = \theta_{\tau_{j} \, i} = \alpha_{1} + \alpha_{j} \mathrm{I}_{j} + \gamma A_{i} + \zeta_{j} \mathrm{I}_{j} A_{i}  $$

where the I_*j*_,*j*=2,…,*M* are *M*−1 indicator functions for estimation of the baseline function. The same indicator functions are used to model time-varying covariate effects.

The estimate of the difference in RMST through follow-up according to binary covariate *A* is given by the step function

14 $$ \mathrm{D}(\tau_{j}) = \Delta(\tau_{j} | A) = \gamma + \zeta_{j} \mathrm{I}_{j}  $$

changing value at each time selected for the computation of the pseudo-values.

The variance of D(*τ*_*j*_) can be estimated for each *τ*_*j*_ from model results as in standard GEE modelling (though, as already said, this may be slightly conservative). For example the variance of D(*τ*_*j*_), can be computed using the robust sandwich variance-covariance matrix of the coefficients *γ* and *ζ*_*j*_ and an *M*-dimensional basis vector, with 1 in the first and *j* positions and 0 otherwise.:

$$\begin{aligned} \left(\begin{array}{ccccc} 1 & \ldots & 1 & \ldots & 0\\ \end{array}\right) \left(\begin{array}{cccc} \mathrm{V}(\widehat{\gamma}) & \ldots & \text{Cov}(\widehat{\gamma}, \widehat{\zeta}_{j}) & \ldots\\ \ldots & \ldots & \ldots & \ldots \\ \ldots & \text{Cov}(\widehat{\zeta}_{j-1}, \widehat{\zeta}_{j}) & \mathrm{V}(\widehat{\zeta}_{j}) &\ldots \\ \ldots & \ldots & \ldots & \ldots \\ \ldots & \ldots & \ldots & \mathrm{V}(\widehat{\zeta}_{M}) \\ \end{array}\right) \left(\begin{array}{c} 1 \\ \vdots \\ 1 \\ \vdots \\ 0 \end{array}\right) \end{aligned} $$

The function D(*τ*_*j*_) can also be obtained by incorporating a smooth spline basis into the regression model. In this case, without loss of generality, considering just two basis functions (for ease of notation) B_1_(*t*) and B_2_(*t*) and an intercept, to model the baseline RMST through time, the regression model can be written as:

15 $$\begin{array}{*{20}l} \text{RMST}(t \mid A_{i}) & = \delta_{0} + \delta_{1} \mathrm{B}_{1}(t) + \delta_{2} \mathrm{B}_{2}(t) + \gamma A_{i} +  \\ & \qquad \zeta_{1} \mathrm{B}_{1}(t) A_{i} + \zeta_{2} \mathrm{B}_{2}(t) A_{i}  \end{array} $$

and the difference in RMST curves is given by the smooth function:

16 $$ \mathrm{D}(t) = \Delta(t | A) = \gamma + \zeta_{1} \mathrm{B}_{1}(t) + \zeta_{2} \mathrm{B}_{2}(t)  $$

The robust variance at time *t* of the estimate $\widehat {\mathrm {D}}(t), V(\widehat {\mathrm {D}}(t))$ can be computed as:

$$\begin{aligned} \left(\begin{array}{ccc} 1 & \mathrm{B}_{1}(t) & \mathrm{B}_{2}(t)\\ \end{array}\right) \left(\begin{array}{ccc} \mathrm{V}(\widehat{\gamma}) & \text{Cov}(\widehat{\gamma}, \widehat{\zeta}_{1}) & \text{Cov}(\widehat{\gamma}, \widehat{\zeta}_{2})\\ \text{Cov}(\widehat{\gamma}, \widehat{\zeta}_{1}) & \mathrm{V}(\widehat{\zeta}_{1}) & \text{Cov}(\widehat{\zeta}_{1}, \widehat{\zeta}_{2})\\ \text{Cov}(\widehat{\gamma}, \widehat{\zeta}_{2}) & \text{Cov}(\widehat{\zeta}_{1}, \widehat{\zeta}_{2}) & \mathrm{V}(\widehat{\zeta}_{2}) \end{array}\right) \left(\begin{array}{c} 1 \\ \mathrm{B}_{1}(t) \\ \mathrm{B}_{2}(t) \end{array}\right) \end{aligned} $$

Based on the asymptotic normality of the model estimates, the asymptotic pointwise 95% confidence interval of R(*t*) is given by $[\mathrm {D}_{lo}(t); \mathrm {D}_{up}(t)] = \widehat {\gamma } + \widehat {\zeta }_{1} \mathrm {B}_{1}(t) + \widehat {\zeta }_{2} \mathrm {B}_{2}(t) \pm 1.96 \sqrt {\mathrm {V}(\widehat {\mathrm {D}}(t))}$.

The confidence region for the curve, i.e. the simultaneous 95% confidence interval of D(*t*), can be obtained by adopting the approach developed by Hothorn et al. (2008) [20]. In order to ensure a coverage probability of at least 95% for the entire curve, an appropriate critical value *u*_95*%*_ must be chosen instead of the 1.96. The value can be chosen such that P(*t*_*max*_≤*u*_95*%*_)=95*%*, where:

17 $$ t_{max} = \text{sup}_{t\in[a,b]}\frac{[\widehat{\gamma} + \widehat{\zeta}_{1} \mathrm{B}_{1}(t) + \widehat{\zeta}_{2} \mathrm{B}_{2}(t) ] - \mathrm{D}(t)}{\sqrt{\mathrm{V}(\widehat{\mathrm{D}}(t))}}  $$

where the limits of the interval [*a*,*b*] span the follow-up time of interest or, more strictly, corresponds to the minimum and maximum times used to compute pseudo-values. In order to compute the *u*_95*%*_ value, the supremum of the function can be obtained using an equally spaced grid of time points [*a*≤*t*_1_≤*t*_1_≤⋯≤*t*_*k*_=*b*]. The obtained value should be sufficiently close to the true value and this approach makes it possible to use standard software for the calculation [29]. The function glht from the package multcomp [20] can be used to compute the confidence band.

## Model selection

An empirical solution for model selection, including for example the number of spline bases, is to use the quasi information criterion (QIC) [30]:

18 $$ \text{QIC} = -2 \text{QL} + 2 \; \text{tr}(\mathbf{N}^{-1} \mathbf{V})  $$

where **N** is the naïve variance estimate, considering independent values, while **V** is the robust variance estimate and QL is the quadratic quasi likelihood for the model with pseudo-values $\sum _{i=1}^{N} \sum _{j=1}^{M} [\mathbf { \widehat { \theta }}_{\tau _{j} i} - \widehat {g^{-1}(\theta _{\tau _{j} \, i})} ]^{2}$. This is in line with the use of pseudo-residuals for the evaluation of the goodness of fit used in [31]. When the selection does not regard the working correlation structure, the trace could simply be replaced by twice the number of model parameters. More details are reported in the supplementary material [see Additional file 1]. More principled approaches are emerging in literature [32], and will hopefully improve also the possibilities of model selection.

## Results

### Simulation

#### Use of multiple restriction times

The use of a vector of pseudo-values at a grid of *M* time points is standard practice in applications of pseudo values involving multi state models. In applications with RMST only a single time point, i.e. the restriction time, was used. In the applications presented here we are using a vector of restriction times, and therefore multiple pseudo-values per subject, in order to estimate the difference between RMST curves through follow-up time together with a confidence band. In this simulation we want to investigate the behaviour of the model estimated with multiple pseudo-values per subject by comparing it to the standard application of pseudo values with a single restriction time. For comparison, the approach proposed by Tian et al. (2014) [12] based on weighted equations is also used. In particular, we use the same simulation design proposed in [25].

Weibull distributed life times were generated with scale parameter *λ*_*i*_= exp(*β*_*b*_*Z*_*i*_) and shape parameter *δ*=0.5, 1 or 2. Here, *Z*_*i*_ is binary with *Pr*(*Z*_*i*_=1)=0.5 and *β*_*b*_=0 or 1. Exponential censoring at 25% was superimposed and the restricted mean life time at *τ* was estimated for values of *τ* at the *p*^*th*^ percentile when *β*_*b*_=0, i.e. *τ*=(−*log*(1−*p*))^1/*δ*^ for *p*=0.75 and 0.9. The true value of the restricted mean is

$$\begin{aligned} \int_{0}^{\tau} \exp(- \lambda t^{\delta}) dt = \frac{1}{\delta} \lambda^{-1/\delta} \left[ \Gamma(\frac{1}{\delta}, 0) - \Gamma(\frac{1}{\delta}, \lambda (-log(1-p))) \right] \end{aligned} $$

where *Γ*(*a*,*x*) is the incomplete gamma function. The baseline RMST is therefore,

$\beta _{0} = \frac {1}{\delta } \left [ \Gamma (\frac {1}{\delta }, 0) - \Gamma (\frac {1}{\delta }, (-log(1-p))) \right ]$, while the Z effect, i.e. the difference in RMST between *Z*=1 and *Z*=0 is given by

$$\begin{aligned} \beta_{1}=\frac{1}{\delta} \exp^{-1/\delta} \left[ \Gamma\left(\frac{1}{\delta}, 0\right) - \Gamma\left(\frac{1}{\delta}, \exp(1) (-log(1-p))\right) \right] - \beta_{0}. \end{aligned} $$

For the standard model with pseudo-values proposed in [25] and for the model of Tian [12], *β*_0_ and *β*_1_ correspond to the intercept and to the coefficient of *Z*. For the model with a vector of pseudo values, 16 times were selected at quantiles of the failure time distribution, starting from the minimum until the 99^*th*^ percentile, and the pseudo-values for each subject were calculated. Natural splines were used to estimate the baseline RMST and an interaction between splines bases and *Z* was used to estimate the curve D(*t*). The value of baseline RMST and of R(*t*) at time *τ* are then calculated, corresponding to *β*_0_ and *β*_1_.

Each combination was replicated 1000 times. Simulations in which the last simulated event time was less than *τ* were excluded. This happened in an important number of times with setting *δ*=0.5 and *β*_*b*_=1 (72 times with *p*=0.75 and 472 times with *p*=0.90 when *N*=250 and 325 times with *p*=0.90 when *N*=1000). Also with setting *δ*=1 and *β*_*b*_=1 this happened 140 times with *p*=0.90 when *N*=250 and 95 times with *p*=0.90 when *N*=1000. In these two settings it happened also that the last restriction time of the model estimated using a vector of pseudo-values (the last restriction time is placed at the 99% percentile of the failure time distribution) was less than *τ* (*δ*=0.5 and *β*_*b*_=1: 22 times with *p*=0.75 and 988 times with *p*=0.90 when *N*=250 and 1000 times with *p*=0.90 when *N*=1000; *δ*=1 and *β*_*b*_=1 this happened 279 times with *p*=0.90 when *N*=250 and 38 times with *p*=0.90 when *N*=1000). Results are shown in Table 1. The biases were everywhere quite small for all the methods compared, with the exception of the model with the vector pseudo-values in setting *δ*=0.5 and *β*_*b*_=1, especially for the 90^*th*^ percentile. This was due to the fact that for the direct model with a vector of pseudo-values the estimates at *τ* were obtained in extrapolation. The number of spline bases was chosen in each simulated data with *QIC* in a range between 3 and 12. However, results were not changing fixing the degrees of freedom to 3 in each simulation (not shown).

**Table 1:** Bias of the regression models estimated with pseudo-values using a single restriction time at *τ* (PV scalar) or multiple restriction times at quantiles of failure time distribution (PV vector), and with the approach of Tian et al, (2004). For PV vector 16 pseudo values were used in each setting. QIC was used to select the degrees of freedom of the splines (from a minimum of 3 to a maximum of 12). Two different sample size were considered (250 and 1000 with 25% censoring)

				P=0.75			P=0.90		
		*δ*	*β*_*b*_	PV scalar	Tian	PV vector	PV scalar	Tian	PV vector
N=250	Baseline	0.5	0	-0.001	-0.001	-0.001	0.003	0.003	0.026
		0.5	1	0.000	0.001	0.014	0.026	0.025	0.137
		1	0	0.000	0.000	-0.001	0.000	0.000	0.011
		1	1	-0.003	-0.003	0.002	-0.005	-0.005	0.004
		2	0	-0.001	-0.001	-0.001	-0.001	-0.001	-0.001
		2	1	-0.001	-0.001	0.001	-0.001	-0.001	0.001
	Z effect	0.5	0	0.001	0.001	0.001	0.003	0.003	0.003
		0.5	1	0.000	-0.001	-0.006	0.043	0.036	0.181
		1	0	0.000	0.000	0.000	0.000	0.000	0.000
		1	1	-0.003	-0.003	-0.013	-0.004	-0.005	-0.002
		2	0	0.002	0.002	-0.002	0.002	0.002	0.002
		2	1	0.002	0.002	-0.002	0.002	0.002	0.002
N=1000	Baseline	0.5	0	0.005	0.005	0.002	-0.003	-0.003	0.023
		0.5	1	0.001	0.001	0.014	0.001	0.001	0.084
		1	0	0.0003	0.0003	-0.002	0.0001	0.0001	0.013
		1	1	-0.001	-0.0005	0.005	-0.001	-0.001	0.002
		2	0	0.0003	0.0003	0.0002	0.0003	0.0003	0.0002
		2	1	0.0003	0.0003	0.003	0.0003	0.0003	0.003
	Z effect	0.5	0	0.001	0.001	0.001	0.005	0.005	0.005
		0.5	1	0.002	0.001	-0.007	0.005	0.004	0.120
		1	0	0.001	0.001	0.001	0.0002	0.0002	0.0005
		1	1	-0.0002	-0.0002	-0.011	-0.001	-0.001	-0.009
		2	0	0.0002	0.0002	-0.0002	0.0002	0.0002	0.0002
		2	1	0.0004	0.0004	-0.0003	0.0004	0.0004	0.0003

#### RMST curve

**Fig. 1:**
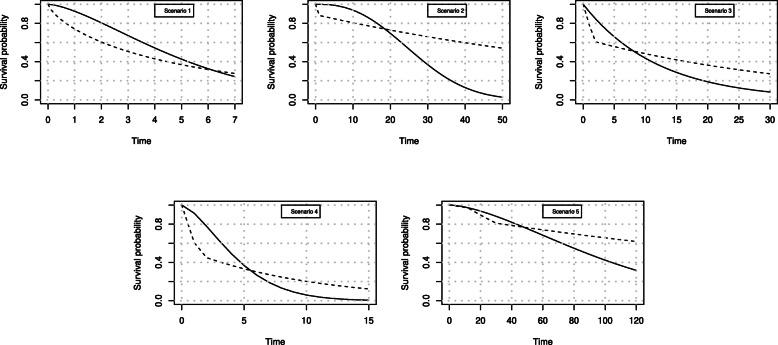
Solid and dotted lines represent the survival curves simulated according to the 5 different scenarios described in RMST curve section

In order to examine the proposed method to estimate the confidence band for the RMST difference curve, different simulation scenarios were performed. The simulated survival functions are represented in Fig. 1.

For RMST curve simulations, event times were simulated according to the following specifications:

- Scenario 1: (1) Weibull with parameters (0.18;1.5) and (2) Weibull with parameters (0.20;0.75).
- Scenario 2: (1) Weibull with parameters (2.5;30) and (2) piecewise exponential with $\lambda =0.125 \, I(t<1) + 0.01 \, I(t\geqslant 1)$.
- Scenario 3:(1) exponential with *λ*=1/12 and a piecewise exponential with $\lambda =0.25 \, I(t<2) + \frac {1}{35} \, I(t\geqslant 2)$.
- Scenario 4:(1) Weibull with parameters (1.5;5) and (2) piecewise exponential with $\lambda =0.5 \, I(t<1.5) + 0.1 \, I(t\geqslant 1.5)$.
- Scenario 5:(1) Weibull with parameters (1.6;110) and (2) piecewise exponential with $\lambda =0.0025 \, I(t<12) + 0.01 \, I(12 \leqslant t <30) + 0.003 \, I(t\geqslant 30)$. This scenario is similar to the data from the EBMT-NMAM2000 study.

For all simulations a 20% random uniform censoring was considered. The first scenario regards a typical situation in which the proportional hazards assumption is not reasonable and the curves are crossing at the end of the considered follow-up. The scenarios from 2 to 4 are taken from [33] where different testing procedures were compared in the presence of crossing survival curves. The crossing is at different probability levels.

The fifth simulation scenario is used to mimic the crossing of survival curves found in the NMAM2000 trial. In this scenario, the two survival curves are practically superimposed at the beginning, then separate and then cross.

For each simulation the RMST difference curve was calculated, using the KM estimator, together with its 95% confidence band, according to the nonparametric method [11]. The curve and the 95% confidence band were also estimated using pseudo-values and GEE regression. To calculate the confidence band with pseudo-values a grid of 10 or more, equally spaced, time points was used. In general the result is quite stable using 10 or more time points. To evaluate the coverage, it was checked if the band included the true RMST difference value, for all the time points of the grid. The average length of the band was also computed together with the average bias in the estimate. Results are reported in Table 2. For the pseudo-value model, 16 time points were considered, at quantiles of the event time distribution.

**Table 2:** Comparison of the results obtained with the non-parametric estimator and the regression model on pseudo values. Sixteen pseudo values are used in each setting. QIC was used to select the degrees of freedom of the splines (from a minimum of 4 to a maximum of 12). The different scenarios are described in Fig. 1. Two different sample size are considered (200 and 400 per group with 20% censoring)

		Non Parametric			Pseudo-Values		
	Scenario	Bias	Coverage	Length	Bias	Coverage	Length
200	1	0.095	0.941	0.643	0.096	0.941	0.612
	2	0.719	0.931	4.297	0.717	0.943	4.425
	3	0.702	0.937	4.634	0.704	0.952	4.524
	4	0.172	0.928	1.140	0.172	0.938	1.115
	5	1.434	0.973	9.886	1.446	0.954	9.499
400	1	0.069	0.946	0.464	0.069	0.928	0.437
	2	0.502	0.939	3.138	0.503	0.937	3.225
	3	0.502	0.948	3.539	0.506	0.929	3.398
	4	0.125	0.944	0.866	0.125	0.945	0.839
	5	1.033	0.965	7.143	1.038	0.942	6.931

For each scenario, natural splines with different degrees of freedom were used varying from 4 to 12. QIC was used to select the degrees of freedom in each simulated data set. Boundary knots were set to the minimum and maximum time used for pseudo-value calculation.

Results from simulations show that the regression model with pseudo-values has results comparable with those of the non-parametric estimators. The coverage of the band is good and approximates quite closely the desired 95%.

### Applications

#### The CSL1 trial in liver cirrhosis

The CSL1 trial was already analysed in [25] with pseudo-values considering both mean and restricted mean survival time, with restriction at 5 years. The randomized trial studied the effect of prednisone on survival in patients with liver cirrhosis [34]. An interesting finding was that only patients without ascites seemed to benefit from the treatment.

The reanalysis presented here aims to compare three different approaches to the analysis of restricted mean: the method based on a single pseudo value at a specified *τ*; the weighted regression of Tian [12] with restricted mean at a specified *τ*; the method with multiple pseudo values per patient at multiple restriction times, useful to estimate the RMST difference curve. All regression models were fitted considering an adjustment by age as in [25]. To compare the results from the different approaches, the first two methods were applied multiple times, varying the restriction time from 1 until 9 years. Regarding the last method, 16 pseudo-times, at quantiles of the failure time distribution (minimum 0.01 and maximum 9.90 corresponding to 99 percentile), were used, obtaining 16 pseudo-values for each patient. Then a regression model with identity link function and interaction between ascites and treatment was estimated. All effects were time dependent as required by the identity link. Two degrees of freedom were used for the spline function according to QIC. The QIC of the model with interaction between ascites and treatment was lower than that of the model without (19497 vs 19775).

The model can be written as:

19 $$\begin{array}{*{20}l} \text{RMST}(t) & = \delta_{0} + \delta_{1} \mathrm{B}_{1}(t) + \delta_{2} \mathrm{B}_{2}(t)  \\ & \quad + \gamma_{1} Asc + \gamma_{2} A + \gamma_{3} Age + \gamma_{4} Asc \times A  \\ & \quad + \zeta_{1} \mathrm{B}_{1}(t) \times Asc + \zeta_{2} \mathrm{B}_{2}(t) \times Asc  \\ & \quad + \zeta_{3} \mathrm{B}_{1}(t) \times A + \zeta_{4} \mathrm{B}_{2}(t) \times A. \\ & \quad + \zeta_{5} \mathrm{B}_{1}(t) \times Age + \zeta_{6} \mathrm{B}_{2}(t) \times Age  \\ & \quad + \zeta_{7} \mathrm{B}_{1}(t) \times Asc \times A + \zeta_{8} \mathrm{B}_{2}(t) \times Asc \times A  \end{array} $$

where B_1_(*t*),B_2_(*t*) are the 2 spline bases, *Asc* is equal to 1 for patients with ascites and 0 otherwise, *A* is 1 for patents treated with prednisone and 0 otherwise, and the variable *Age* is measured in years. The interest here is in the estimated difference between treatments of the RMST curves, according to the presence of ascites and adjusted by age that is given by:

$$\begin{array}{*{20}l} \Delta(t \mid Asc) & = \gamma_{2} + \gamma_{4} Asc + \zeta_{3} \mathrm{B}_{1}(t) + \zeta_{4} \mathrm{B}_{2}(t)\\ & \quad + \zeta_{7} \mathrm{B}_{1}(t) \times Asc + \zeta_{8} \mathrm{B}_{2}(t) \times Asc \end{array} $$

**Fig. 2:**
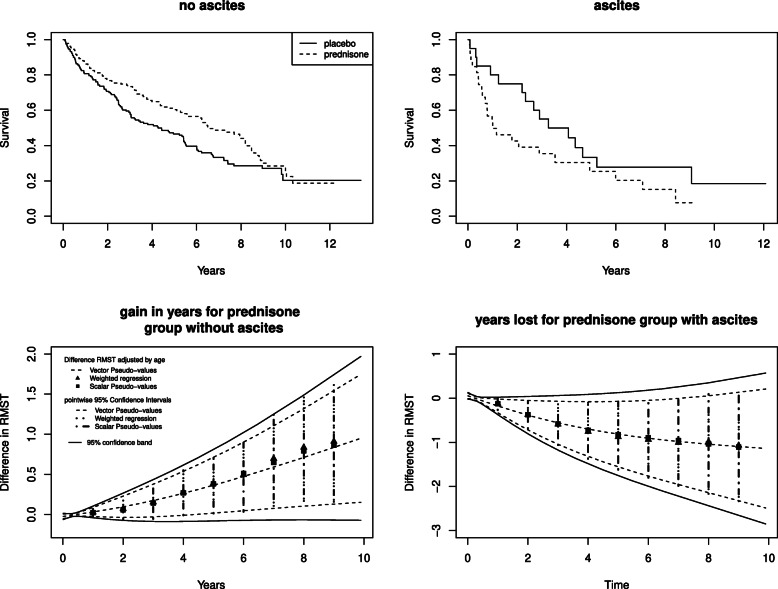
CSL1 Trial in Liver Cirrhosis. On the top the Kaplan-Meier survival curves for the ascites and no ascites groups of patients. On the bottom the difference of RMST in the two groups for different restriction times and direct modelling approaches. The small vertical lines on the *x* axis represent the times used to calculate the pseudo-values. All estimates are adjusted by age (years)

The Kaplan-Meier curves in the ascites and no ascites groups and the difference between treatments of the RMST curves, estimated with multiple pseudo values with their 95% point-wise confidence intervals and bands, are reported in Fig. 2.

Moreover, the estimates obtained applying multiple times pseudo-value regression with a single restriction time, varying *τ* from 1 until 9 years, are reported. The model with *τ*=5 is the one used in [25].

The same procedure was applied for the model of Tian [12] using the R function rmst2 from the package survRM2 [14]. In this case it was also necessary to perform two separate regressions in the ascites and non ascites groups, adjusted by age. Results from the three different modelling approaches are quite similar with the advantage, for our method, of making possible the estimation of the simultaneous confidence bands for the curves.

#### Colon cancer trial

We used colon cancer data, available in the *survivalR* package [35], from a trial of adjuvant chemotherapy for colon cancer comparing Levamisole and Levamisole plus 5-FU (a chemotherapy agent) ([36, 37]). The re-analysis presented by Eng and Seagle (2017) [38] explored the complex pattern of interaction between age and treatment using RMST. In fact it appeared that age was significantly associated with relapse in the Levamisole plus 5-FU arm but not the Levamisole alone arm. Looking at how RMST (restricted at 60 months) varied with age it appeared that for patients who were younger than 50 years there was no difference between treatments, whereas for those older than 50 years there was up to a 12-month delay in relapse. The analysis was repeated with 16 pseudo-times, at quantiles of the failure time distribution. A regression model with identity link function and a time-varying interaction between age and treatment was estimated. Four degrees of freedom were used for the natural spline function according to QIC. Moreover, the model with interaction between age and treatment had a lower QIC than the model without (865558 vs 869017) and was selected.

The model can be written as:

20 $$ \begin{aligned} \text{RMST}(t) & = \delta_{0} + \delta_{1} \mathrm{B}_{1}(t) + \ldots + \delta_{4} \mathrm{B}_{4}(t) \\ & \quad + \gamma_{1} A + \gamma_{2} Age + \gamma_{3} Age \times A \\ & \quad + \zeta_{1} \mathrm{B}_{1}(t) \times Age + \ldots + \zeta_{4} \mathrm{B}_{4}(t) \times Age \\ & \quad + \zeta_{5} \mathrm{B}_{1}(t) \times A + \ldots + \zeta_{8} \mathrm{B}_{4}(t) \times A. \\ & \quad + \zeta_{9} \mathrm{B}_{1}(t) \times Age \times A + \ldots + \zeta_{12} \mathrm{B}_{4}(t) \times Age \times A \end{aligned}  $$

where B_1_(*t*),…,B_4_(*t*) are the 4 spline bases, *A* is 1 for patents treated with Levamisole plus 5-FU and 0 otherwise, and the variable *Age* is measured in years.

The difference between treatments of the RMST curves is therefore varying with age and given by:

21 $$\begin{array}{*{20}l} \Delta(t \mid Age) & = \gamma_{1} + \gamma_{3} Age  \\ & \quad + \zeta_{5} \mathrm{B}_{1}(t) + \ldots + \zeta_{8} \mathrm{B}_{4}(t). \\ & \quad + \zeta_{9} \mathrm{B}_{1}(t) \times Age + \ldots + \zeta_{12} \mathrm{B}_{4}(t) \times Age  \end{array} $$

**Fig. 3:**
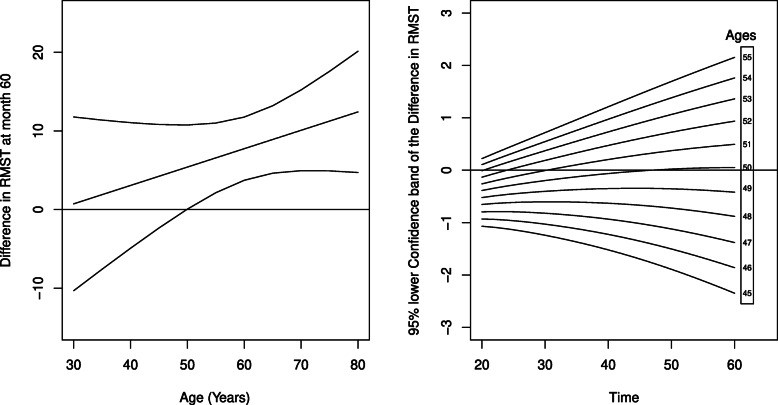
Colon cancer. Left: difference in restricted mean time to relapse between the Levamisole and Levamisole plus 5-FU at time 60 for different ages. The lower limit of the 95% pointwise confidence interval is above 0 for ages greater than 50. Right: For different ages, the lower 95% confidence band through follow-up is shown

The analysis of the difference between treatments of the RMST curves confirms previous findings. The right panel of Fig. 3 shows *Δ*(*t*∣*Age*) with *t*=60, reproducing the results obtained in [38]. It is possible to see how the RMST difference between treatments, restricted at 60 months, is varying with age. The difference is significant for ages greater than 50 as shown by the 95% confidence interval.

The difference between treatments of the RMST can be estimated for all the follow-up times to evaluate the association with age. The left panel of Fig. 3 reports the lower limit of the 95% confidence band of the curve through follow-up for different values of age. It is possible to observe how the lower limit is above 0 for all the follow-up times when considering ages greater than 53.

#### EBMT-NMAM2000 study

The third application refers to the NMAM2000 trial comparing tandem autologous/reduced intensity conditioning allogeneic transplantation (auto+allo) to autologous transplantation alone (auto) on an intent-to-treat basis. The analysis and the corresponding clinical considerations are published in [39] while those presented here are illustrative considerations for the statistical methods presented.

**Fig. 4:**
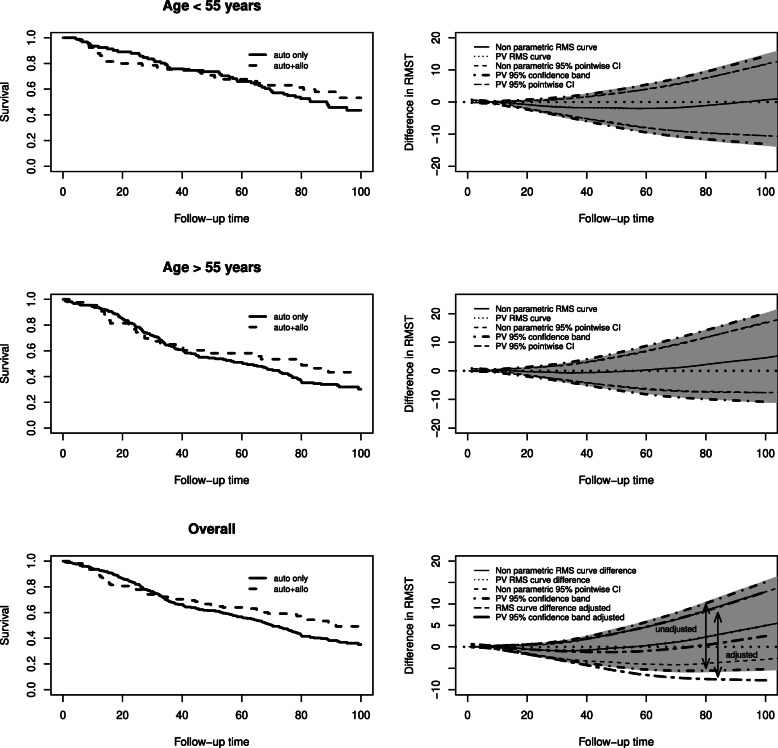
Multiple Myeloma: Overall survival curves (left panels) and differences in RMST between the treatments estimated non-parametrically (continuous) and with the pseudo values (dot) (right panels). Top and middle panels refer to patients with less and more than 55 years, respectively. The bottom panel shows the overall sample of patients. In right panels, the grey areas correspond to the 95% confidence band estimated with the non-parametric method while the thick dotdashed lines correspond to the 95% confidence bands estimated with pseudo-values. The figures also show the point-wise 95% confidence interval for the non-parametric method (dashed) and for the pseudo-values method (longdashed). The bottom right panel shows also the difference in RMST adjusted for age (twodash)

The overall survival probability curves are reported in the top right panel of Fig. 4. The curves have a similar pattern for the first year, then they separate with auto+allo group having more events than auto group, later the curves cross at about 33 months where auto+allo seems superior to the auto group. Considering the crossing of the curves, based on a clinical rationale, a comparison at a fixed point in time was used, namely 96 months (13% difference, p=0.03). The same comparison using RMST at 96 months did not provide evidence of difference (4.4 months difference, p=0.27). When the analysis was stratified by age group (using 55 as cutoff), the survival curves for younger patients showed that the initial disadvantage was, unexpectedly, worse than in elderly.

The analysis of the RMST curve was performed with 16 pseudo-times, at quantiles of the failure time distribution. Two regression models with identity link function, with and without a time-varying interaction between age and treatment, were estimated. Three degrees of freedom were used for the natural spline function. The model with interaction had a lower QIC than the model without (1644218.3 vs 1645363.9). The interaction was not statistically significant (Wald test p=0.97, df=4), while the effect of age was significant (p=0.03, df=4).

The entire RMST curves are reported in the bottom right panels of Fig. 4, for the stratified and the overall sample.

The curves show the difference in the area under the overall survival curve between the auto-allo and auto groups. Considering the overall sample, the difference was near 0 at the beginning, then becomes negative turning definitely positive afterwards by crossing the x axis. The upper and the lower limits of the confidence band never crossed the x axis. The curve for younger patients was, for almost the entire follow-up, below 0, showing no advantage for the auto+allo group. The curve for older patients started to show an advantage for auto+allo group at about 5 years.

The unadjusted and the age adjusted difference between RMST were reported in the bottom right panel of Fig. 4. It is possible to see how the adjusted curve crossed the x axis later in time with respect to the unadjusted estimate.

## Discussion

The use of HR in clinical studies is generally accepted as a useful measure of association. Notwithstanding this, the debate about the use of HR is always active, especially because its use is strictly tied with the Cox regression model and the assumption of proportional hazards. In fact, especially with long follow-up length, the tenability of this assumption becomes more questionable [10]. Moreover, concerns about the clinical usefulness of the HR are always present, as it is difficult to translate an HR in terms of clinical benefit. In general, as no single measure can be useful in all circumstances, it is advisable not to simply rely on the HR to quantify the association in time to event analysis.

Many alternatives have been proposed in the literature. For example, the difference (or ratio) in survival probabilities at a specific time point, or the difference (or ratio) of RMST at a specific time point could be taken into consideration. These proposals have the obvious drawback that a single time point should be selected for the analysis. In some circumstances, as in the application presented on multiple myeloma, a clinically relevant time horizon is present but this is not always the case.

In this perspective, the proposal to look at how the difference of RMST varies through follow-up is particularly appealing and dates back to the work of Royston and Parmar (2011) [8]. The main caveat when looking at the entire curve is that it would be appropriate to resort to a confidence band instead of the point-wise confidence limit. This was the object of a recent proposal based on the Kaplan-Meier estimator [11].

In this work, direct modelling of RMST through a regression model using pseudo-values with time dependent effects, was proposed with the advantage of including different covariates, thus proving an adjusted RMST difference and a confidence band. The method is in good agreement with the estimates obtained by direct regression models fixing one restriction time. Moreover, the method is flexible enough to reproduce the results of the model-free method when no covariates are considered. We showed the method in several examples where age plays an important role and must be considered in the analysis.

In principle, other flexible regression models could be used for the same purpose. In practice, the estimation based of pseudo-values can rely completely on standard available software for the confidence band calculation. Moreover through the use of pseudo values it should be possible to extend the approach to the competing risks setting considering the cause-specific years lost as described in [15]. One drawback is that it is necessary to choose how many time points to use for pseudo-values calculations and how to space them. Although this aspect should be further investigated, it seems that varying the number of time points does not alter substantially the results. On the other hand, at present, only the non-parametric estimator of the RMST curve [11] does not require to specify the time points for the curve estimation. The analysis of the curve through all the regression models considered here is, in fact, a collection of analyses at different restriction times.

## Supplementary Information

**Additional file 1** An illustrative example with different methods for RMST estimation.

**Additional file 2** R code for figures 2 and 3.

## Data Availability

Source R code to perform the computations of The CSL1 trial in liver cirrhosis and Colon cancer trial sections is available in the supplementary material [see Additional file 2]. The code uses the publicly available datasets colon [36, 37] from survival R package [40] (https://CRAN.R-project.org/package=survival) and CSL from the data accompanying the book of Andersen and Skovgaard [41] (http://staff.pubhealth.ku.dk/~linearpredictors/datafiles/Csl.csv).

## References

[1] Marubini E, Valsecchi MG (1995). Analysing Survival Data from Clinical Trials and Observational Studies.

[2] Spruance SL, Reid JE, Grace M, Samore M (2004). Hazard ratio in clinical trials. Antimicrob Agents Chemother.

[3] Hernan MA (2010). The hazards of hazard ratios. Epidemiology.

[4] Martinussen T, Vansteelandt S, Andersen PK (2020). Subtleties in the interpretation of hazard contrasts. Lifetime Data Anal.

[5] Perperoglou A, Keramopoullos A, van Houwelingen HC (2007). Approaches in modelling long-term survival: an application to breast cancer. Stat Med.

[6] Greenhalgh T, Howick J, Maskrey N. Evidence based medicine: a movement in crisis?BMJ. 2014;348. 10.1136/bmj.g3725. https://www.bmj.com/content/348/bmj.g3725. 10.1136/bmj.g3725 PMC4056639 24927763

[7] Uno H, Claggett B, Tian L, Inoue E, Gallo PP, Miyata T, Schrag D, Takeuchi M, Uyama Y, Zhao L, Skali H, Solomon S, Jacobus S, Hughes M, Packer M, Wei L (2014). Moving beyond the hazard ratio in quantifying the between-group difference in survival analysis. J Clin Oncol.

[8] Royston P, Parmar MK (2011). The use of restricted mean survival time to estimate the treatment effect in randomized clinical trials when the proportional hazards assumption is in doubt. Stat Med.

[9] Klein JP, Moeschberger ML (2003). Survival Analysis Techniques for Censored and Truncated Data, 2nd Edn.

[10] Royston P, Parmar MK (2013). Restricted mean survival time: an alternative to the hazard ratio for the design and analysis of randomized trials with a time-to-event outcome. BMC Med Res Methodol.

[11] Zhao L, Claggett B, Tian L, Uno H, Pfeffer MA, Solomon SD, Trippa L, Wei LJ (2016). On the restricted mean survival time curve in survival analysis. Biometrics.

[12] Tian L, Zhao L, Wei LJ (2014). Predicting the restricted mean event time with the subject’s baseline covariates in survival analysis. Biostatistics.

[13] Klein JP, Gerster M, Andersen PK, Tarima S, Perme MP (2008). Sas and r functions to compute pseudo-values for censored data regression. Comput Methods Programs Biomed.

[14] Uno H, Tian L, Horiguchi M, Cronin A, Battioui C, Bell J. survrm2: Comparing restricted mean survival time. R package version. 2005;1.

[15] Andersen PK (2013). Decomposition of number of life years lost according to causes of death. Stat Med.

[16] Andersen PK, Klein JP, Rosthøj S (2003). Generalised linear models for correlated pseudo-observations, with applications to multi-state models. Biometrika.

[17] Diggle PJ, Liang KY, Zeger SL (1994). Analysis of Longitudinal Data.

[18] Liang K-Y, Zeger SL (1986). Longitudinal data analysis using generalized linear models. Biometrika.

[19] Overgaard M, Parner E, Pedersen J (2017). Asymptotic theory of generalized estimating equations based on jack-knife pseudo-observations. Ann Statist.

[20] Hothorn T, Bretz F, Westfall P (2008). Simultaneous inference in general parametric models. Biom J.

[21] Karrison T (1987). Restricted mean life with adjustment for covariates. J Am Stat Soc.

[22] Zucker DM (1988). Restricted mean life with covariates: Modification and extension of a useful survival analysis method. J Am Stat Soc.

[23] Scheike TH, Martinussen T (2006). Dynamic Regression Models for Survival Data.

[24] R Core Team (2021). R: A Language and Environment for Statistical Computing.

[25] Andersen PK, Hansen MG, Klein JP (2004). Regression analysis of restricted mean survival time based on pseudo-observations. Lifetime Data Anal.

[26] Klein JP, Andersen PK (2005). Regression modeling of competing risks data based on pseudovalues of the cumulative incidence function. Biometrics.

[27] Ambrogi F, Biganzoli E, Boracchi P (2008). Estimates of clinically useful measures in competing risks survival analysis. Stat Med.

[28] Freireich EJ, Gehan EA, Frei E, Schroeder LR, Wolman IJ, Anbari R, Burgert EO, Mills SD, Pinkel DP, Selawry OS, Moon JH, Gendel BR, Spurr CL, Storrs RC, Haurani FI, Hoogstraten B, Lee SL (1963). The effect of 6-mercaptopurine on the duration of steroid-induced remissions in acute leukemia: A model for evaluation of other potentially useful therapy. Blood.

[29] Bretz F, Hothorn T, Westfall P (2011). Multiple Comparisons Using R.

[30] Pan W (2001). Akaike’s information criterion in generalized estimating equations. Biometrics.

[31] Perme MP, Andersen PK (2008). Checking hazard regression models using pseudo-observations. Stat Med.

[32] Pavlič K, Martinussen T, Andersen PK (2019). Goodness of fit tests for estimating equations based on pseudo-observations. Lifetime Data Anal.

[33] Li H, Han D, Hou Y, Chen H, Chen Z (2015). Statistical inference methods for two crossing survival curves: a comparison of methods. PLoS ONE.

[34] Christensen E, Schlichting P, Andersen PK, Fauerholdt L, Juhl E, Poulsen H, Tygstrup N (1985). A therapeutic index that predicts the individual effects of prednisone in patients with cirrhosis. Gastroenterology.

[35] Therneau TM, Grambsch PM (2000). Modeling Survival Data: Extending the Cox Model.

[36] Moertel CG, Fleming TR, Macdonald JS, Haller DG, Laurie JA, Goodman PJ, Ungerleider JS, Emerson WA, Tormey DC, Glick JH (1990). Levamisole and fluorouracil for adjuvant therapy of resected colon carcinoma. N Engl J Med.

[37] Moertel CG, Fleming TR, MacDonald JS, Haller DG, Laurie JA, Tangen CM, Ungerleider JS, Emerson WA, Tormey DC, Glick JH, Veeder MH, Maillard JA (1991). Fluorouracil plus levamisole as an effective adjuvant therapy after resection of stage ii colon carcinoma: a final report. Annals of Internal Med.

[38] Eng KH, Seagle BL (2017). Covariate-adjusted restricted mean survival times and curves. J Clin Oncol.

[39] Gahrton G, Iacobelli S, Björkstrand B, Hegenbart U, Gruber A, Greinix H, Volin L, Narni F, Carella AM, Beksac M, Bosi A, Milone G, Corradini P, Schönland S, Friberg K, van Biezen A, Goldschmidt H, de Witte T, Morris C, Niederwieser D, Garderet L, Krvger N, and for the EBMT Chronic Malignancies Working Party Plasma Cell Disorders Subcommittee (2013). Autologous/reduced-intensity allogeneic stem cell transplantation vs autologous transplantation in multiple myeloma: long-term results of the ebmt-nmam2000 study. Blood.

[40] Therneau TM (2020). A package for survival analysis in r. R package version.

[41] Andersen PK, Skovgaard LT (2010). Regression with Linear Predictors.

