# Supplementary material for: Analyzing differences between restricted mean survival time curves using pseudo-values
Source: BMC Med Res Methodol. 2022 Mar 18;22:71. doi: 10.1186/s12874-022-01559-z (PMC8931966; doi:10.1186/s12874-022-01559-z)
Supplement: Supplementary file 1 — Additional file 1 An illustrative example with different methods for RMST estimation. [file 12874_2022_1559_MOESM1_ESM.pdf]

ADDITIONAL FILE 1

F. AMBROGI, S. IACOBELLI, P.K. ANDERSEN

An illustrative example

For illustration of the pseudo-values method, we use the classical Freireich data [1]. We compare the results obtained using pseudo-values with two other approaches, namely the flexible regression method of Royston and Parmar [2], and the direct regression method of Tian et al. [3], based on weighted estimating equations. Freireich's data deal with a trial in childhood leukemia comparing length of remission in (paired) groups treated with 6-MP or placebo. The data are available in [4] and were used also in [5] for illustrative purposes. In the placebo group there was no censoring.

Supplementary table S1 shows the observed times in remission and the pseudo-values for the RMST with $\tau = 23$ for the 6-MP treatment group together with the placebo group. For censored time we observe large pseudo-values while pseudo-values for uncensored times are smaller than the actually observed time in remission. A very simple illustrative code for the calculation of pseudo-values is the following:

```
library(survival)
library(bpcp)
library(pseudo)
library(rstpm2)
library(survRM2)
library(timereg)
data("leuk2")

 km <- survfit(Surv(time, status) ~ 1, data=leuk2)
 n <- nrow(leuk2)
 PV.f <- NULL
 for(i in 1:n){
   kmi <- survfit(Surv(time, status) ~ 1, data=leuk2[-i,])
   PVi <- n * survival:::survmean(km, rmean=23)$matrix[5] -
(n-1)*survival:::survmean(kmi, rmean=23)$matrix[5]
   PV.f <- c(PV.f, PVi)
 }

 mean(PV.f)
[1] 13.06564
```

For calculating pseudo-values it is easier to use the fuction `pseudomean` from the `R` package `pseudo` [6].

Note that the non parametric estimate of the restricted mean survival time at $\tau = 23$ is $\int_0^\tau \widehat{\mathrm{S}}(t)dt = 13.1$, as the average of the pseudo-values.

```
km <- survfit(Surv(time, status) ~ 1, data=leuk2)
survival:::survmean(km, rmean=23)

$matrix
   records      n.max    n.start     events     *rmean *se(rmean)     median    0.95LCL  
 42.000000  42.000000  42.000000  30.000000  13.065641   1.249938  12.000000   8.000000  2

$end.time
[1] 23
```

The mean value of the pseudo-values for the 6-Mp group is 17.7, while for the placebo group is 8.4. The RMST calculated as $\int_0^\tau \widehat{\mathrm{S}}(t)dt$ is equal to 17.9 in the 6-MP group and 8.7 in the placebo group.

6-MP		Placebo	
Observed Remission Length (weeks)	pseudo-values	Observed Remission Length (weeks)	pseudo-values
1	1.00	6	6.00
1	1.00	6	6.00
2	2.00	6	6.00
2	2.00	6+	16.79
3	3.00	7	6.65
4	4.00	9+	18.73
4	4.00	10	9.35
5	5.00	10+	19.16
5	5.00	11+	20.12
8	7.69	13	11.81
8	7.69	16	15.42
8	7.69	17+	23.58
8	7.69	19+	23.58
11	10.00	20+	23.58
11	10.00	22	22.29
12	10.60	23	23.95
12	10.60	25+	23.95
15	14.22	32+	23.95
17	16.62	32+	23.95
22	22.29	34+	23.95
23	23.95	35+	23.95

Supplementary table S1. Observed times in remission and the pseudo-values for the RMST with $\tau = 23$ for the 6-MP treatment group and the placebo group

A regression model with pseudo-values as the dependent variable as a function of the treatment group

$$\text{RMST}(t) = \gamma_0 + \gamma_1 treatment$$

gives the following result:

```
PV.model <- geeglm(PV.f ~ I(treatment=="6-MP"),
                   data=leuk2,
                   id = 1:n, scale.fix=TRUE,
                   family=gaussian(link = "identity"), corstr="independence")
summary(PV.model)

Call:
geeglm(formula = PV.f ~ I(treatment == "6-MP"), family = gaussian(link = "identity"),
    data = leuk2, id = 1:n, corstr = "independence", scale.fix = TRUE)

 Coefficients:
                          Estimate Std.err Wald Pr(>|W|)
```

```
(Intercept)                    8.38    1.38 37.1  1.1e-09 ***
I(treatment == "6-MP")TRUE     9.37    2.05 21.0  4.7e-06 ***
---
Signif. codes:  0 ‘***’ 0.001 ‘**’ 0.01 ‘*’ 0.05 ‘.’ 0.1 ‘ ’ 1

Correlation structure = independence
Scale is fixed.

Number of clusters:   42  Maximum cluster size: 1
```

The RMST for the placebo group is estimated equal to 8.38 while γ_1 estimates the difference of RMST(τ=23) between 6-MP and placebo groups.

The analysis can be performed using the approach of Royston and Parmar [2] using flexible parametric survival models. The estimate of the restricted mean survival time at time τ is obtained using a regression model on the log cumulative hazard function. This estimate is then transformed into the survival function and integrated over the interval $(0, \tau)$. Standard errors can be estimated using the bootstrap or the delta method. In the following code the function `stpm2`, [7], is used to estimate the log cumulative hazard function, then the `predict` function provides the estimate of the RMST.

```
 flexsurv.rmst <- stpm2(Surv(time,status)~treatment, data=leuk2,df=3)
 summary(flexsurv.rmst)

 Maximum likelihood estimation

Call:
stpm2(formula = Surv(time, status) ~ treatment, data = leuk2,
    df = 3)

Coefficients:
                        Estimate Std. Error z value   Pr(z)
(Intercept)               -5.068      0.972   -5.21 1.9e-07 ***
treatmentplacebo           1.681      0.422    3.99 6.7e-05 ***
nsx(log(time), df = 3)1    3.051      0.671    4.55 5.4e-06 ***
nsx(log(time), df = 3)2    6.228      1.703    3.66 0.00026 ***
nsx(log(time), df = 3)3    3.271      0.511    6.40 1.6e-10 ***
---
Signif. codes:  0 ‘***’ 0.001 ‘**’ 0.01 ‘*’ 0.05 ‘.’ 0.1 ‘ ’ 1

-2 log L: 213
 predict(flexsurv.rmst, newdata=data.frame(treatment="6-MP", time=23),
   type="rmst", se.fit=TRUE)

    Estimate lower upper
1       18    15  20.9
```

```
 predict(flexsurv.rmst, newdata=data.frame(treatment="placebo", time=23),
   type="rmst", se.fit=TRUE)

    Estimate lower upper
1     8.46     6  10.9
```

The methods of Zucker [8] is implemented in the R function `restricted.residual.mean` from the `timereg` package [9]. The result is reported below:

```
leuk2$treat<-1*(leuk2$treatment=="6-MP")
out<-cox.aalen(Surv(time,status)~prop(treat),data=leuk2,max.timepoint.sim=NULL,resample.ii
coxrm <- restricted.residual.mean(out, tau=23, x=rbind(0, 1),iid=1)
summary(coxrm)
  mean   se
  8.99 1.21
 17.90 1.46
```

The last method illustrated here is that of Tian and colleagues [3] based on inverse probability weighted estimating functions. The implementation is in package `survRM2` [10]. When there are no covariates the function uses the non parametric RMST estimator $\int_0^\tau \widehat{\mathrm{S}}(t)dt$, having estimates equal to those reported above.

```
 ipcw.model <- rmst2(leuk2$time, leuk2$status, leuk2$treatment=="6-MP", tau=23)
 ipcw.model

The truncation time: tau = 23  was specified.

Restricted Mean Survival Time (RMST) by arm
              Est.   se lower .95 upper .95
RMST (arm=1) 17.91 1.55     14.87      21.0
RMST (arm=0)  8.67 1.38      5.97      11.4
```

The implementation in SAS `proc rmstreg` [11] returns the following results (reference 6-MP group):

```
proc rmstreg data=one tau=23;
class treat;
model T*status(0) = treat / link = linear method = ipcw(strata=treat);
run;

Parameter   Stima Err St   lower upper Chi-quad Pr > ChiQuadr
```

```
Intercept   17.7663 1.5919 14.6462 20.8865 124.55 <.0001
treatment   -9.0997 2.1051 -13.2256 -4.9737 18.69 <.0001
```

The SAS `proc rmstreg` can perform the analysis using the method of Tian [3] and also using pseudo-values. For the sake of comparison of the results across different software, we report below the analysis with `proc rmstreg` with the use of pseudo-values. We use as reference the placebo group for direct comparison with the result obtained in `R`.

```
proc rmstreg data=one tau=23;
class treat (ref = '0');
model T*status(0) = treat / link = linear method = pv;
run;

Parameter   Stima Err St   lower upper Chi-quad Pr > ChiQuadr
Intercept   8.3819 1.3763 5.6844 11.0795 37.09 <.0001
treatment   9.3675 2.0462 5.3570 13.3780 20.96 <.0001
```

The summary of the results from the different approaches is reported in the Supplementary table S2.

	6MP	placebo
pseudo-values	17.8 (14.8 - 20.7)	8.38 (5.7 - 11.1)
proc rmstreg	17.8 (14.6 - 20.9)	8.7 (6.0 - 11.4)
stpm2	18.0 (15.0 - 20.9)	8.5 (6.0 - 10.9)
cox.aalen	17.9 (15.0 - 20.8)	9.0 (6.7 - 11.4)

Supplementary table S2. Comparison of the results from the regression model with pseudo-values, the model of Tian and colleagues [3] obtained using `SAS proc rmstreg`, the flexible regression of Royston and Parmar [2] using `stpm2 R` function, and the method of Zucker [8] from the `R` function `cox-aalen`.

Considering pseudo-values, it is possible for example, to do the calculations at $\tau = 15$ and $\tau = 23$. This will result in two pseudo-values for each subject. Stacking the data, it is possible to perform the analysis simultaneously for the two restriction times. Below you can see the code for pseudo-values calculation and the first 20 rows of the stacked data.

```
 bv <- data.frame()
for(j in c(15, 23)){
  pseudo = pseudomean(time=leuk2$time, event=leuk2$status, tmax=j)
  a <- cbind(leuk2, pseudo = pseudo, id=1:nrow(leuk2), tau=rep(j, nrow(leuk2)))
  bv <- rbind(bv, a)
}
bv <- bv[order(bv$id),]
head(bv, 20)

  time status treatment pair treat pseudo id tau
1      1      1   placebo    1     0   1.00  1  15
43     1      1   placebo    1     0   1.00  1  23
2     10      1      6-MP    1     1   9.70  2  15
44    10      1      6-MP    1     1   9.35  2  23
3     22      1   placebo    2     0  15.19  3  15
45    22      1   placebo    2     0  22.29  3  23
4      7      1      6-MP    2     1   6.79  4  15
46     7      1      6-MP    2     1   6.65  4  23
5      3      1   placebo    3     0   3.00  5  15
47     3      1   placebo    3     0   3.00  5  23
6     32      0      6-MP    3     1  15.19  6  15
48    32      0      6-MP    3     1  23.95  6  23
7     12      1   placebo    4     0  11.58  7  15
49    12      1   placebo    4     0  10.60  7  23
8     23      1      6-MP    4     1  15.19  8  15
50    23      1      6-MP    4     1  23.95  8  23
9      8      1   placebo    5     0   7.82  9  15
51     8      1   placebo    5     0   7.69  9  23
10    22      1      6-MP    5     1  15.19 10  15
52    22      1      6-MP    5     1  22.29 10  23
```

The regression model with the estimates obtained are reported in the code below.

```
vectorPV <- geeglm(pseudo ~ as.factor(tau) * I(treatment=="6-MP"),
                   data=bv, id = id,
                   scale.fix=TRUE, family=gaussian(link = "identity"),
                   corstr="independence")
summary(vectorPV)

Call:
geeglm(formula = pseudo ~ as.factor(tau) * I(treatment == "6-MP"),
```

```
    family = gaussian(link = "identity"), data = bv, id = id,
    corstr = "independence", scale.fix = TRUE)

 Coefficients:
                                             Estimate Std.err  Wald Pr(>|W|)
(Intercept)                                     7.783   1.054 54.49  1.6e-13 ***
as.factor(tau)23                                0.599   0.533  1.26     0.26
I(treatment == "6-MP")TRUE                      5.090   1.299 15.34  9.0e-05 ***
as.factor(tau)23:I(treatment == "6-MP")TRUE     4.278   0.997 18.39  1.8e-05 ***
---
Signif. codes:  0 ‘***’ 0.001 ‘**’ 0.01 ‘*’ 0.05 ‘.’ 0.1 ‘ ’ 1

Correlation structure = independence
Scale is fixed.

Number of clusters:   42  Maximum cluster size: 2
```

where the intercept estimates RMST for the placebo group at $\tau = 15$ (non parametric estimate equal to 7.86), the second coefficient estimates the difference between RMST for the placebo group between times $\tau = 15$ and $\tau = 23$ (non parametric estimate for the placebo group at $\tau = 23$ equal to 8.67), the third coefficient estimates the difference between 6-MP and placebo groups at time $\tau = 15$ (non parametric estimate for the 6-MP group at $\tau = 15$ equal to 12.92), and the interaction estimates the difference between RMST for the 6-MP group between times $\tau = 15$ and $\tau = 23$ (non parametric estimate for the 6-MP group at $\tau = 23$ equal to 17.91).

Quasi-likelihood function for pseudo-observations

The presented application of pseudo-values models for the RMST tries to evaluate the difference between RMST curves through follow-up times. To obtain a smooth function we propose to use regression splines. The spline complexity, i.e. the number of knots, may be chosen on a clinical/application ground. For example if only mild non linearities are expected a spline with few knots may be sufficient. To select the complexity on the basis of a goodness-of-fit statistic we may try to borrow from some criteria specifically designed for generalized estimating equations (GEE).

The pseudo-values are calculated at a set of M selected time points, the pseudo times $(\tau_1, \ldots, \tau_M)$, as

$$\hat{\theta}_{i,j} = n \int_0^{\tau_j} \hat{S}(\tau_j) - (n-1) \int_0^{\tau} \hat{S}_{-i}(\tau_j) \tag{0.1}$$

where $\hat{S}_{-i}(\tau_j)$ is the leave-one-out Kaplan-Meier estimator at time τ_j obtained by deleting the subject i.

The vector $\boldsymbol{\theta}_i = (\hat{\theta}_{i,1}, \ldots, \hat{\theta}_{i,M})$ is the vector of pseudo-values for subject i, while $g[\theta_{\tau_j\, i}] = \beta^T \mathbf{Z}$ is the regression model on pseudo-values (equation 12 in the main text). The model matrix $\boldsymbol{Z}_i$, is obtained replicating M times the covariate vector of subject i. According to the estimating equations (equation 6 in the main text),

the model with pseudo-values is based on a Gaussian quasi likelihood (with a scale parameter fixed to 1):

$$ql(\boldsymbol{\beta}) = -\frac{1}{2}\sum_{i=1}^{n}(\hat{\boldsymbol{\theta}}_i - \boldsymbol{g}^{-1}(\beta^T\mathbf{Z}))^T(\hat{\boldsymbol{\theta}}_i - \boldsymbol{g}^{-1}(\beta^T\mathbf{Z})). \tag{0.2}$$

In fact, according to chapter 4 of [12], the Gaussian quasi likelihood is given by $-\frac{1}{2}\sum(y-\mu)^2$.

A goodness of fit measure was proposed for GEE by Pan [13], a quasi information criterion (QIC):

$$\mathrm{QIC} = -2\mathrm{QL} + 2\ \mathrm{tr}(\mathbf{N}^{-1}\mathbf{V}) \tag{0.3}$$

where $\mathbf{N}$ is the naïve variance estimate, considering independent values, while $\mathbf{V}$ is the robust variance. This criterion was proposed to select from different correlation structures, while if the goal is selecting covariates, or, such as in our case, the number of knots of the spline, the QIC_u can be used, [12], substituting the trace with $2p$, where p is the number of parameters in the model, like in the original AIC. This is a pragmatic solution to the problem of model selection while other approaches, more tailored to the specificities of pseudo-values, are emerging in literature, [14], and will hopefully bridge the gap in this framework.

References

[1] Freireich, E.J., Gehan, E.A., Frei, E., Schroeder, L.R., Wolman, I.J., Anbari, R., Burgert, E.O., Mills, S.D., Pinkel, D.P., Selawry, O.S., Moon, J.H., Gendel, B.R., Spurr, C.L., Storrs, R.C., Haurani, F.I., Hoogstraten, B., Lee, S.L.: The effect of 6-mercaptopurine on the duration of steroid-induced remissions in acute leukemia: A model for evaluation of other potentially useful therapy. Blood **21**, 699–716 (1963)

[2] Royston, P., Parmar, M.K.: The use of restricted mean survival time to estimate the treatment effect in randomized clinical trials when the proportional hazards assumption is in doubt. Stat Med **30(19)**, 2409–2421 (2011)

[3] Tian, L., Zhao, L., Wei, L.J.: Predicting the restricted mean event time with the subject's baseline covariates in survival analysis. Biostatistics **15(2)**, 222–233 (2014)

[4] Klein, J.P., Moeschberger, M.L.: Survival Analysis Techniques for Censored and Truncated Data, 2nd Edn. Springer, New York (2003)

[5] Andersen, P.K., Hansen, M.G., Klein, J.P.: Regression analysis of restricted mean survival time based on pseudo-observations. Lifetime Data Anal **10(4)**, 335–350 (2004)

[6] Perme, M.P., Gerster, M.: Pseudo: Computes Pseudo-Observations for Modeling. (2017). R package version 1.4.3. https://CRAN.R-project.org/package=pseudo

[7] Liu, X.-R., Pawitan, Y., Clements, M.: Parametric and penalized generalized survival models. Statistical Methods in Medical Research **27**(5), 1531–1546 (2018)

[8] Zucker, D.M.: Restricted mean life with covariates: Modification and extension of a useful survival analysis method. Journal of the American Statistical Society **93**, 702–709 (1988)

[9] Scheike, T.H., Martinussen, T.: Dynamic Regression Models for Survival Data. Springer, N.Y (2006)

[10] Uno, H., Tian, L., Horiguchi, M., Cronin, A., Battioui, C., Bell, J.: survrm2: Comparing restricted mean survival time. R package version **1** (2005)

[11] Guo, C., Liang, Y.: Analyzing restricted mean survival time using sas/stat. In: SAS Conference Proceedings: SAS Global Forum 2019. SAS Institute Inc., Cary NC (2019). Paper SAS3013-2019

[12] Hardin, J.W., Hilbe, J.M.: Generalized Estimating Equations. Chapman and Hall/CRC, N.Y (2002)

[13] Pan, W.: Akaike's information criterion in generalized estimating equations. Biometrics **57**, 120–125 (2001)

[14] Pavlič, K., Martinussen, T., Andersen, P.K.: Goodness of fit tests for estimating equations based on pseudo-observations. Lifetime Data Anal **25**, 189–205 (2019)
