# Supplementary material for: Analyzing differences between restricted mean survival time curves using pseudo-values
Source: BMC Med Res Methodol. 2022 Mar 18;22:71. doi: 10.1186/s12874-022-01559-z (PMC8931966; doi:10.1186/s12874-022-01559-z)
Supplement: Supplementary file 2 — Additional file 2 R code for figures 2 and 3. [file 12874_2022_1559_MOESM2_ESM.pdf]

Additional file 2

```
Manuscript Title: Analyzing differences between restricted mean survival
time
curves using pseudo-values
Authors: Federico Ambrogi, Simona Iacobelli, Per Kragh Andersen
Code by: Federico ambrogi (federico.ambrogi@unimi.it)

This file presents the computer code in R for the manuscript "Analyzing
differences between restricted mean survival time curves using pseudo-
values".
The code reproduces figures 2 and 3 of the manuscript.

Required R packages: pseudo, splines, multcomp, survival, geepack, survRM2.


sessionInfo()

R version 4.0.5 (2021-03-31)
Platform: x86_64-apple-darwin17.0 (64-bit)
Running under: macOS Catalina 10.15.7


# Code for: Analyzing differences between restricted mean survival time
# curves using pseudo-values
#-----------------------------------------------------------------------
# PURPOSE: Illustrate the estimate of the difference of RMST between
# two treatments using pseudo-values DATE: 22 Jul 2020
#-----------------------------------------------------------------------
#
# The CSL1 trial was already analysed in Andersen et al (2004) with
pseudoobservations
# considering both mean and restricted mean survival time, with
# restriction at 5 years.
# The randomized trial studied the effect of prednisone on
# survival in patients with liver cirrhosis (Christensen et al, 1985). An
interesting
# finding was that only patients without ascites seemed to benefit from the
# treatment. The reanalysis presented here aims to compare three different
# approaches to the analysis of restricted mean:
# 1) the method based on pseudo value at a single point in time,
# 2) the weighted regression of Tian et al (2014) with restricted mean at a
specified time
# 3) the method with pseudo values with multiple restriction times used to
estimate the RMST curve.

rm(list = ls())

library(pseudo)
library(splines)
library(multcomp)
library(geepack)
library(survival)
library(survRM2)

# Read the CSL data
csl <-
read.csv2("http://staff.pubhealth.ku.dk/~linearpredictors/datafiles/Csl.csv
",
```

```
                 sep = ";",
                 dec = ".",
                 header=TRUE,
                 colClasses =
                     c("factor","factor","numeric",
                       "factor","factor","factor","numeric",
                       "numeric","numeric","factor"),
                 na.strings="."
)

# CSL1 was a randomized clinical trial where, in the period 1962-69, 488
# patients with liver cirrhosis were treated with either the active drug
# prednisone (251 patients) or placebo (237 patients). The purpose of
# the trial was to evaluate the effect of treetment on survival.  After
# randomization patients were followed to either death, drop-out or end
# of study (September 1974): 142 prednisone patients and 150 placebo
patients
# died. The survival times for the remaining patients are
# right-censored.
#
# The data file includes the following variables:
#
# ID = patient id. (integer, range 1-561)
# DC = 1, if failure 0, if censoring
# DAYS = survival time in days from randomization
# TMENT = 0, if prednisone 1, if placebo
# SEX = 0, if female, 1, if male
# ASC = 0, if no ascites, 1, if some, 2, if moderate or marked
# AGE (in years, range 17-80)
# PRO(thrombin) (in % of normal, range 12-135)
# ACE(tylcholinesterase) (in micromoles/min/ml, range 26-659)
# INFL(ammation in liver connective tissue) = 0, if none, 1, if slight, 2,
if
# moderate, 3, if severe


## followup in years
csl$years <- csl$days/365.25
csl$asc2 <- 1*(csl$asc != 0)
head(csl)

par(mfrow=c(2,2))
plot(survfit(Surv(years, dc == 1) ~ tment, data = csl, subset = asc==0),
lty=1:2, main="no ascites", xlab="Years", ylab = "Survival")
legend("topright", legend=c("placebo", "prednisone"), lty=1:2, cex=.8)
plot(survfit(Surv(years, dc == 1) ~ tment, data = csl, subset = asc==1),
lty=1:2, main="ascites", xlab="Years", ylab = "Survival")

## We would like the placebo group to be the reference group
csl$tment <- relevel(csl$tment, ref = "1")
table(csl$dc)

model1 <- coxph(Surv(days, dc==1) ~ asc2 + tment + asc2:tment + age, data =
csl)
summary(model1)
```

```
## -------------------------------------------------------------------------
## pseudo values with multiple restriction times and confidence band
## -------------------------------------------------------------------------

D <- csl
######
NPV <- 16
cutoffs = c(min(D$years[D$dc==1]), quantile(D$years[D$dc==1], seq(.01, .99,
by=.98/NPV )))
summary(csl$years[csl$dc==1])
cutoffs
bv <- data.frame()
#compute the pseudo-observations:
for(j in 1:length(cutoffs)){
    pseudo = pseudomean(time=D$years, event=D$dc==1,tmax=cutoffs[j])
    a <- cbind(D, pseudo = pseudo, id=1:nrow(D), time=rep(cutoffs[j],
nrow(D)))
    bv <- rbind(bv, a)
}
bv <- bv[order(bv$id),]
head(bv)

BK <- c(min(D$years[D$dc==1]), cutoffs[length(cutoffs)])
basis <- ns(bv$time, df=2, Boundary.knots=BK)
vectorPV <- geeglm(pseudo ~ basis * (asc2 * tment + age),
                   data=bv, id = id, scale.fix=TRUE, family=gaussian(link =
"identity"), corstr="independence")
summary(vectorPV)
QIC(vectorPV)

# It is possible to try different spline node numbers and calculate QIC.
# 2 df is in correspondence of the minimum QICu

## -------------------------------------------------------------------------
## treatment effect in no Ascites group
## -------------------------------------------------------------------------

xa <- cbind(1, predict(basis, cutoffs), asc = 0, tment = 0, age=40,
0,predict(basis, cutoffs)*0,
            predict(basis, cutoffs)*0, predict(basis, cutoffs)*40,
predict(basis, cutoffs)*0)
xb <- cbind(1, predict(basis, cutoffs), asc = 0, tment = 1, age=40, 0,
predict(basis, cutoffs)*0,
            predict(basis, cutoffs)*1, predict(basis, cutoffs)*40,
predict(basis, cutoffs)*0)
ma <- nrow(xa)
mb <- nrow(xb)
X <- xb - xa
X
m <- nrow(X)
K <- as.matrix(X)
gmod_ci <- try(confint(glht(vectorPV, linfct = K)))
gmod_ci

se.t = diag(X %*% vcov(vectorPV) %*% t(X))
upper.p <-  X %*% coef(vectorPV) - 1.96 * sqrt(se.t)
lower.p <-  X %*% coef(vectorPV) + 1.96 * sqrt(se.t)
pred <- (X %*% coef(vectorPV))

plot(cutoffs, pred, lty=2, type="l", ylim=c(-.2, 2), xlab="Years",
ylab="Difference in RMST",
```

```
     main="gain in years for prednisone \n group without ascites")
lines(cutoffs, upper.p, lty=2)
lines(cutoffs, lower.p, lty=2)

lines(cutoffs, gmod_ci$confint[,"lwr"], lty=1)
lines(cutoffs, gmod_ci$confint[,"upr"], lty=1)

# 2) the weighted regression of Tian et al. (2014) with restricted mean at
a specified time
# ranging from 1 to 9 years
csl.temp <- subset(csl, subset= asc2==0)
for(tau in 1:9){
tian <- rmst2(csl.temp$years, csl.temp$dc==1, csl.temp$tment, tau = tau,
covariates = csl.temp$age, alpha = 0.05)
lines(c(tau, tau), tian$RMST.difference.adjusted[2,5:6], lty=3, lwd=2)
points(tau, tian$RMST.difference.adjusted[2,1], pch=17)
}

# 1) Pseudo value at a single point in time, ranging from 1 to 9 years

for(tau in 1:9){
b <- data.frame()
#compute the pseudo-observations:
    pseudo = pseudomean(time=D$years, event=D$dc==1,tmax=tau)
    #arrange the data
    b <- cbind(D, pseudo = pseudo, id=1:nrow(D), time=rep(tau, nrow(D)))
    b <- b[order(b$id),]
#head(b)
scalar <- geese(pseudo ~ asc2 * tment + age,
                data=b, id = id, scale.fix=TRUE, family=gaussian,
scale.value = 1,
                mean.link="identity", corstr="independence")
summary(scalar)
lines(c(tau, tau), c(scalar$beta[3] - 1.96 * sqrt(scalar$vbeta[3,3]),
scalar$beta[3] + 1.96 * sqrt(scalar$vbeta[3,3])), lty=4, lwd=2)
points(tau, scalar$beta[3], pch=15)
}
legend(0,1.5, legend=c("Vector Pseudo-values", "Weighted regression",
"Scalar Pseudo-values"), lty=c(2, 3, 4),
       cex=.6, title = "pointwise 95% Confidence Intervals", lwd=c(1,2,2),
bty="n")
legend(0,2, legend=c("Vector Pseudo-values", "Weighted regression", "Scalar
Pseudo-values"), lty=c(2, NA, NA), pch=c(NA, 17, 15),
       cex=.6, title = "Difference RMST adjusted by age", bty="n")
legend(0,1, legend=c("95% confidence band"), lty=1, cex=.6, bty="n")

## ---------------------------------------------------------------------------
## treatment effect in Ascites group
## ---------------------------------------------------------------------------

xa <- cbind(1, predict(basis, cutoffs), asc = 1, tment = 0, age=40,
0,predict(basis, cutoffs)*1,
            predict(basis, cutoffs)*0, predict(basis, cutoffs)*40,
predict(basis, cutoffs)*0)
xb <- cbind(1, predict(basis, cutoffs), asc = 1, tment = 1, age=40, 1,
predict(basis, cutoffs)*1,
            predict(basis, cutoffs)*1, predict(basis, cutoffs)*40,
predict(basis, cutoffs)*1)
ma <- nrow(xa)
mb <- nrow(xb)
X <- xb - xa
```

```
X
m <- nrow(X)
K <- as.matrix(X)

gmod_ci <- try(confint(glht(vectorPV, linfct = K)))
gmod_ci

se.t = diag(X %*% vcov(vectorPV) %*% t(X))
upper.p <-  X %*% coef(vectorPV) - 1.96 * sqrt(se.t)
lower.p <-  X %*% coef(vectorPV) + 1.96 * sqrt(se.t)
pred <- (X %*% coef(vectorPV))

plot(cutoffs, pred, lty=2, type="l", ylim=c(-3, 1), xlab="Time",
ylab="Difference in RMST",
     main="years lost for prednisone \n group with ascites")
lines(cutoffs, upper.p, lty=2)
lines(cutoffs, lower.p, lty=2)

lines(cutoffs, gmod_ci$confint[,"lwr"], lty=1)
lines(cutoffs, gmod_ci$confint[,"upr"], lty=1)


csl.temp <- subset(csl, subset= asc2==1)
for(tau in 1:9){
    tian <- rmst2(csl.temp$years, csl.temp$dc==1, csl.temp$tment, tau =
tau, covariates = csl.temp$age, alpha = 0.05)
    lines(c(tau, tau), tian$RMST.difference.adjusted[2,5:6], lty=3, lwd=2)
    points(tau, tian$RMST.difference.adjusted[2,1], pch=17)
}

for(tau in 1:9){
    b <- data.frame()
    #compute the pseudo-observations:
    pseudo = pseudomean(time=D$years, event=D$dc==1,tmax=tau)
    b <- cbind(D, pseudo = pseudo, id=1:nrow(D), time=rep(tau, nrow(D)))
    b <- b[order(b$id),]
    scalar <- geese(pseudo ~ asc2 * tment + age,
                    data=b, id = id, scale.fix=TRUE, family=gaussian,
scale.value = 1,
                    mean.link="identity", corstr="independence")
    summary(scalar)
    se.t = c(1,1) %*% scalar$vbeta[c(3,5), c(3,5)] %*% c(1,1)
    lines(c(tau, tau), c(scalar$beta[3]+scalar$beta[5] - 1.96 * sqrt( se.t
),
                         scalar$beta[3]+scalar$beta[5] + 1.96 *
sqrt(se.t)), lty=4, lwd=2)
    points(tau, scalar$beta[3]+scalar$beta[5], pch=15)
}
par(mfrow=c(1,1))


# ---------------------------------------------------------------------------
# The colon data are available in R in the package survival.
# These are data from a trial of Levamisole vs Levamisole + 5-FU
# chemotherapy agent. There are two records per person, one for recurrence
and one for death.
# The data were reanalyzed in Eng K.H. and Seagle B.L. (2017) Covariate-
Adjusted Restricted Mean Survival Times and Curves
# Journal of Clinical Oncology.
# They show how the treatment effect is dependent on age: for ages less
than 50 there is
```

```
# no benefit in Lev+5Fu vs Lev alone.
# the analysis presented here shows the entire RMST curve for different age
values.
# ----------------------------------------------------------------------------

?colon
# etype:    event type: 1=recurrence,2=death
head(colon)
colon2 <- subset(survival::colon, subset=etype==1 & rx != "Obs")
colon2$months <- colon2$time/30.4167
table(colon2$rx)
colon2$rx <- factor(colon2$rx, levels = c("Lev+5FU", "Lev"))
colon2$rx2 <- ifelse(colon2$rx=="Lev+5FU", 1, 0)

head(colon2)
dim(colon2)

plot(survfit(Surv(months, status) ~ rx2, data = colon2), lty=1:2,
xlab="Days", ylab = "Survival")
survfit(Surv(months, status) ~ rx2, data = colon2)
# all: survfit(formula = Surv(months, status) ~ rx2, data = colon2)
#
# n events median 0.95LCL 0.95UCL
# rx2=0 310    172   38.9    26.2      68
# rx2=1 304    119     NA      NA      NA

summary(coxph(Surv(months, status) ~ rx2, data = colon2))
# Call:
#     coxph(formula = Surv(months, status) ~ rx2, data = colon2)
#
# n= 614, number of events= 291
#
# coef exp(coef) se(coef)     z Pr(>|z|)
# rx2 -0.497     0.608    0.119 -4.17  3.1e-05 ***
#     ---
#     Signif. codes:  0 '***' 0.001 '**' 0.01 '*' 0.05 '.' 0.1 ' ' 1
#
# exp(coef) exp(-coef) lower .95 upper .95
# rx2     0.608       1.64     0.481     0.768
#
# Concordance= 0.563  (se = 0.015 )
# Likelihood ratio test= 17.7  on 1 df,   p=3e-05
# Wald test            = 17.4  on 1 df,   p=3e-05
# Score (logrank) test = 17.7  on 1 df,   p=3e-05

D <- colon2
NPV <- 16
cutoffs = c(min(D$months[D$status==1]), quantile(D$months[D$status==1],
seq(.01, .99, by=.98/NPV )))
cutoffs
bv <- data.frame()
#compute the pseudo-observations:
for(j in 1:length(cutoffs)){
    pseudo = pseudomean(time=D$months, event=D$status==1,tmax=cutoffs[j])
    a <- cbind(D, pseudo = pseudo, id=1:nrow(D), tt=rep(cutoffs[j],
nrow(D)))
    bv <- rbind(bv, a)
}
bv <- bv[order(bv$id),]
head(bv)
```

```
BK <- c(min(D$months[D$status==1]), cutoffs[length(cutoffs)])
basis <- ns(bv$tt, df=3, Boundary.knots=BK)
vectorPV <- geeglm(pseudo ~ basis * (rx2 * age),
                   data=bv, id = id, scale.fix=TRUE,
                   family=gaussian(link = "identity"),
                   corstr="independence")
QIC(vectorPV)
# The minimum QICu is with 4 df

vectorPV0 <- geeglm(pseudo ~ basis * (rx2 + age),
                    data=bv, id = id, scale.fix=TRUE,
                    family=gaussian(link = "identity"),
                    corstr="independence")
QIC(vectorPV0)

# using the glht function let's estimate a confidence band through follow-
up times
# at different values of age

toplot.int <- NULL
AGES <- seq(30,80,5)
ll <- length(AGES)
RT <- seq(20,60, 2)
lt <- length(RT)
for(aa in AGES){
    xa <- cbind(1, predict(basis, RT), rx2 = 0, age=rep(aa, lt), 0*rep(aa,
lt),
                predict(basis, RT)*0, predict(basis, RT)*rep(aa, lt),
                predict(basis, RT)*0*rep(aa, lt))
    xb <- cbind(1, predict(basis, RT), 1, rep(aa, lt), 1*rep(aa, lt),
                predict(basis, RT)*1, predict(basis, RT)*rep(aa, lt),
                predict(basis, RT)*1*rep(aa, lt))

    ma <- nrow(xa)
    mb <- nrow(xb)
    X <- xb - xa
    m <- nrow(X)
    K <- as.matrix(X)

    gmod_ci <- confint(glht(vectorPV, linfct = K))
    temp <- data.frame(pred = gmod_ci$confint[,"Estimate"],
                       low = gmod_ci$confint[,"lwr"],
                       up =  gmod_ci$confint[,"upr"],
                       month = RT,
                       age = rep(aa, lt))

    toplot.int <- rbind(toplot.int, temp)
}

ESplot <- subset(toplot.int, subset= month == 60)
plot(ESplot$age, ESplot$pred, type="l", ylim=c(-15, 25))
lines(ESplot$age, ESplot$low)
lines(ESplot$age, ESplot$up)
abline(h=0)
abline(v=50)

toplot.int <- NULL
AGES <- seq(45,55,1)
ll <- length(AGES)
RT <- seq(20,60, 2)
lt <- length(RT)
```

```
for(aa in AGES){
    xa <- cbind(1, predict(basis, RT), rx2 = 0, age=rep(aa, lt), 0*rep(aa,
lt),
                predict(basis, RT)*0, predict(basis, RT)*rep(aa, lt),
                predict(basis, RT)*0*rep(aa, lt))
    xb <- cbind(1, predict(basis, RT), 1, rep(aa, lt), 1*rep(aa, lt),
                predict(basis, RT)*1, predict(basis, RT)*rep(aa, lt),
                predict(basis, RT)*1*rep(aa, lt))

    ma <- nrow(xa)
    mb <- nrow(xb)
    X <- xb - xa
    m <- nrow(X)
    K <- as.matrix(X)

    gmod_ci <- try(confint(glht(vectorPV, linfct = K)))
    temp <- data.frame(pred = gmod_ci$confint[,"Estimate"],
                       low = gmod_ci$confint[,"lwr"],
                       up =  gmod_ci$confint[,"upr"],
                       month = RT,
                       age = rep(aa, lt))

    toplot.int <- rbind(toplot.int, temp)
}
plot(RT, toplot.int$low[toplot.int$age==50], type="n", ylim=c(-3, 3), xlim
= c(20, 65),
     xlab="Time", ylab="95% lower Confidence band of the Difference in
RMST")
abline(h=0)
for(i in AGES){
    temp <- subset(toplot.int, subset= age==i)
    lines(RT, temp$low, lty=1)
}
text(61, min(toplot.int$low[toplot.int$age==45]), labels="45", cex=.6)
text(61, min(toplot.int$low[toplot.int$age==46]), labels="46", cex=.6)
text(61, min(toplot.int$low[toplot.int$age==47]), labels="47", cex=.6)
text(61, min(toplot.int$low[toplot.int$age==48]), labels="48", cex=.6)
text(61, max(toplot.int$low[toplot.int$age==49]), labels="49", cex=.6)
text(61, max(toplot.int$low[toplot.int$age==50]), labels="50", cex=.6)
text(61, max(toplot.int$low[toplot.int$age==51]), labels="51", cex=.6)
text(61, max(toplot.int$low[toplot.int$age==52]), labels="52", cex=.6)
text(61, max(toplot.int$low[toplot.int$age==53]), labels="53", cex=.6)
text(61, max(toplot.int$low[toplot.int$age==54]), labels="54", cex=.6)
text(61, max(toplot.int$low[toplot.int$age==55]), labels="55", cex=.6)
```
